# Protocol for Evaluation of Robotic Technology in Orthopedic Surgery

**DOI:** 10.1155/2013/194683

**Published:** 2013-09-19

**Authors:** Milad Masjedi, Zahra Jaffry, Simon Harris, Justin Cobb

**Affiliations:** MSk Lab, Charing Cross Hospital, Imperial College London, London W6 8RF, UK

## Abstract

In recent years, robots have become commonplace in surgical procedures due to their high accuracy and repeatability. The Acrobot Sculptor is an example of such a robot that can assist with unicompartmental knee replacement. In this study, we aim to evaluate the accuracy of the robot (software and hardware) in a clinical setting. We looked at (1) segmentation by comparing the segmented data from Sculptor software to other commercial software, (2) registration by checking the inter- and intraobserver repeatability of selecting set points, and finally (3) sculpting (*n* = 9 cases) by evaluating the achieved implant position and orientation relative to that planned. The results from segmentation and registration were found to be accurate. The highest error was observed in flexion extension orientation of femoral implant (0.4 ± 3.7°). Mean compound rotational and translational errors for both components were 2.1 ± 0.6 mm and 3 ± 0.8° for tibia and 2.4 ± 1.2 mm and 4.3 ± 1.4° for the femur. The results from all processes used in Acrobot were small. Validation of robot in clinical settings is highly vital to ensure a good outcome for patients. It is therefore recommended to follow the protocol used here on other available similar products.

## 1. Introduction

In recent years, robots have become commonplace in industry due to their high accuracy and repeatability especially during procedures that require movement that is beyond the human control [1, 2]. As imaging and robotic technology has advanced, there is real potential to use these capabilities in the field of surgery, from planning to performing the procedure. This is especially useful in operations such as unicompartmental knee arthroplasty (UKA) where previous studies have shown the substantial effect of implant position inaccuracy [3–5].

The robotic procedures can be fully controlled (active) [6], can be shared as control or semiactive, where the robot monitors surgeon performance and provides stability and support through active constraint [7], they can be tele-surgical where the surgeon performs the operation from a console distant to operating table [8]. The input to the robot can vary from the actual imaging data of the patient to statistical shape models (SSM) [9] or active shape models (ASM) [10, 11] that are based on a few point estimates of the patient's morphology. The main problem with the latter is that these models are often created based on a normal anatomy dataset, and using them for pathological subjects can be problematic [12]. Audenaert et al. described the estimated accuracy of imageless surgery as poor because of the significant difference between the actual location of the probe during surgery and what is displayed on the navigation platform screen [13].

The Acrobot Sculptor (Stanmore Implants Worldwide Ltd.) is a semiactive robot, uses the computer tomography (CT) data as input, and could assist with bone resection for UKA surgery in a consistent manner to minimise variability [14]; however, the repeatability and accuracy of this robot in clinical settings are yet to be determined. In this study, we have set up various steps to determine the accuracy and repeatability of the Stanmore Sculptor which we believe will also be applicable to a wide range of other available similar products. 

There are a number of processes involved in the use of the Sculptor, as with most robotic systems, with potential for error. Surgeons often use the imaging technology such as computed tomography (CT) to identify the pathology and plan the surgery virtually. During the surgery, a registration process takes place that matches the preoperative plan and imaging data to the patient [15]. This means the transformation between the virtual environment and the patient is known and any points in the plan can be located during the surgery. Results can be affected by both the software and hardware used by the robot [16]. 

The patient's CT scan is often segmented using available commercial software (e.g., Acrobot Modeller for Acrobot Sculptor). This software can generate the surface structure of the specified bones. It is possible to use these three-dimensional (3D) images to diagnose the pathology even though the surface geometry is not accurate or in scale. However, in robotic procedures, the accuracy of these surfaces has a direct influence on the outcome of surgery. This surface model is then loaded onto Acrobot Planner software to carry out preoperative planning. The Sculptor has a cutting burr attached to its three degrees of freedom (DoF) arm which can sculpt the bone based on a predefined plan. A tracking arm is pinned to the bone so that the system is aware of the 3D position of that bone relative to the robot at all times. Following attachment of the bone to the tracking arm, the intraoperative procedure also requires registration of points on the bone surfaces [15].

Other than the validity of the software, potential sources of error which can influence the outcome of the surgery include (1) the inaccuracy in position of sculpting arm or tracking arm (poor calibration), (2) inaccuracy in the registration algorithms to match the CT data to the bone, and finally (3) the robotic control system that constrains the surgeon to resect only on the safe zone area. Additionally, there may be other errors arising from surgeons in charge such as poor fixture of bones to tracking arm or inaccuracies in the use of tools [16].

The accuracy of registration, specifically, is an aspect that remains to be determined. There are a number of methods through which registration can take place, such as use of X-ray or ultrasound [17]. Some systems use fiducial markers in order to register the bone and some use landmarks on the bone such as discrete identifiable points or the ridge line [17]. Each is subject to a certain type of error including fiducial localization and registration error and target registration error [18]. The Acrobot Sculptor uses a mechanical digitizer (a secondary use of the robotic arm) to register the surface, where the tip of the cutter (ball point) is used as a probe which has a 2 mm diameter. As a result of inaccuracy in calibration or radius of the ball point, the captured data can be displaced from the true surface. 

Validation of robot is vital to ensure a good outcome and highlight their value in use with patients [19]. Although there are several technical papers that have talked in detail about the accuracy of registration algorithms and robotic manipulations [20], there is no real simple method to test the accuracy of the robot in a clinical environment, and the main reference point simply remains the manufacturer's information. In this study, we aim to evaluate the above possible cause of errors in a clinical setting. 

## 2. Materials and Methods

In order to evaluate the accuracy of the Acrobot Sculptor, the following steps were taken.

The initial step was determining the accuracy of the segmentation procedure. We compared the segmentation result of Modeller (Stanmore Implants, London, UK) from a single femur using various software used to convert CD data to 3D models. These are Mimics (Materialise, Leuven, Belgium) and Robin 3D (Cavendish Medical, London UK) [14]. These surfaces were then matched together using 3-matic (Materialise, Leuven, Belgium) software and the differences in size were analysed. 

The second step in determining the reliability and reproducibility of the Sculptor was by placing a set of points (using a marker pen) on the dry bone femur that was CT scanned. A total of 45 points were selected randomly on the distal part of the femur, focussed on for medial UKA procedures and four observers used the Sculptor to register these points. The root mean squared (RMS) error of the registration process was recorded for each observer. In order to check the effect of different tracking and sculpting arm positions, the same procedure was repeated by changing the fixation of the femur. The positions mimicked those found in surgical operations for various patients' size or surgeons' preference.

The last step is to measure the accuracy of constraints set at the planning stage during bone resection. A senior surgeon (JPC) was recruited to plan the operation using the Planner Software. Uniglide implants (Corin, Cirencester, UK) were chosen (a size four tibial component and size three femoral component) to restore the natural joint line, incorporating a seven-degree posterior slope in the tibial component. Nine UKAs were implanted on identical dry bone knee models (Imperial knee, Medical Models Company, Bristol, UK) by three experienced users of the Sculptor (three each). The models used were CT-based replicas of a patient's arthritic knee consisting of a capsule, replica ligaments, and muscle tissues. Following implantation, the knee joint was separated from femur and tibia and each bone was individually scanned using the NextEngine Desktop 3D scanner (NextEngine, Santa Monica, CA, USA). Prior to implantation, the implant was painted in white enamel paint to improve pick-up of the laser spot from the scanner on the metal surface.

**Figure 1:**
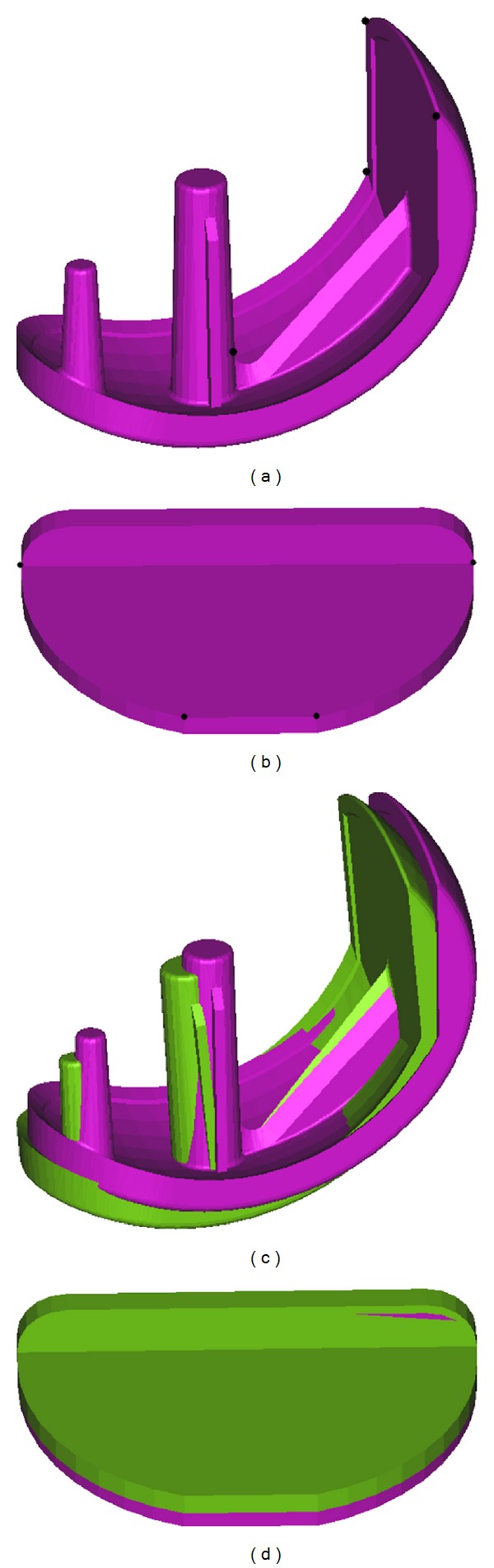
Four points selected on the (a) femoral and (b) tibial implants to construct the local frame of reference. Comparison of the planned versus achieved rotational and translational errors based on the local frame of reference for (c) femoral and (d) tibial implants.

These scans were exported as Stereolithography (STL) files to 3-matic software. The positions of the tibial and femoral components were then compared to those of the ideal plan by recording the coordinates of four points on the planned implants versus the achieved implants (Figure 1). Using MATLAB, a local frame of reference was created using these four points for the achieved implant and was compared to that of the planned in all six DoF. These coordinates were created so that they follow the anatomical frame of reference such that the *x*-, *y*- and *z*-axes correspond to mediolateral, anteroposterior, and superoinferior directions accordingly. The magnitude of translational (a combination of the medial-lateral, anterior-posterior, and superior-inferior directions errors) and rotational (a combination of the axial, flexion-extension, and coronal alignment errors) errors were calculated for each case for both tibial and femoral components.

## 3. Results

The results from segmentation using different software were almost identical. The measurements were performed in 3-matic software, and the difference was far less than 0.5 mm at all points. 

The results for the registration repeatability (second step) showed a consistent mean RMS error were of 0.5 mm while the maximum error among all subjects was found to be 1.8 mm with mean maximum being 1.53 ± 0.2 mm.

**Figure 2:**
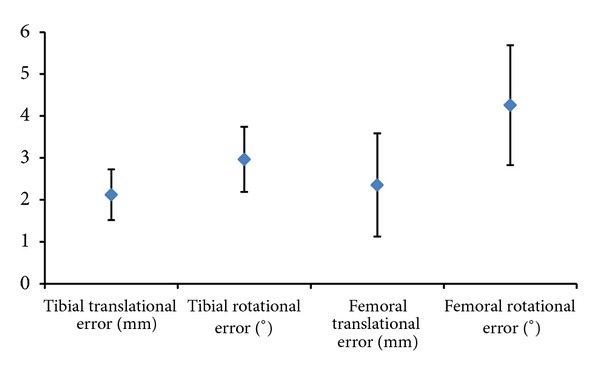
Magnitude of resultant rotational and translation error for tibial and femoral components when compared to planned positions.

The results for implantation are shown in Table 1. Placement of the femoral component in general was more prone to error with a maximum error of 5.3° around the *x*- and *z*-axes. Mean compound rotational and translational errors for tibia component were 2.1 ± 0.6 mm and 3 ± 0.8° and for femoral component were 2.4 ± 1.2 mm and 4.3 ± 1.4° (Figure 2). The highest error was found in rotational elements for both components. 

## 4. Discussion

In this study, we set protocols that make it possible to evaluate robot's accuracy in house, which include testing both software and hardware and are applicable to a variety of similar products on the market. Robotics technology can improve surgical outcomes by providing the surgeon with the greatest amount of accuracy and precision regardless of long surgical training [21]. The robot gives the surgeon more control in terms of the position and alignment of the tools. The accompanied software can also assist in the planning of the surgery and also during the operation by supplying information on direction and amount of cut by enabling the surgeon to visualise these on the screen. These robots are prone to errors both systematic and those due to the operator. Validating the accuracy of image guided surgery is therefore an important issue that needs to be addressed. 

The initial step was determining the accuracy of the segmentation procedure. There are numerous techniques available to create the bone surface from CT images. In lack of any available straight forward method, in this study we used comparative validation and found almost identical data using different software. For the second part we evaluated the landmarks that create the frame of reference. In this study, we found that placement of landmarks can be inaccurate. It is therefore important for surgeons to rely on their own experience for planning the procedure as well as the suggested values given by software for the implant position. 

In this study, rotational error was found to be the highest source of error in both femoral and tibial components. This we believe is actually not only depends on the cut made by robot but also, when the implant is hammered into the plastic bone. This is on especially important with use of plastic bone, as it is possible to deform this under higher loads which may increase the error seen here. Nevertheless, these errors were accepted, far superior to what has been reported for conventional surgeries [14, 22], and similar to other robots available. For example, Dunbar et al. [22] found that the MAKO robot's mean RMS errors for the tibia were 1.4 mm and 2.6° and for the femur 1.2 mm and 2.1°. Furthermore, the ranges found are within the safe range reported by Biomet [23].

We recognise the inherent limitations of our study, one of which is the use of dry replica bones rather than patients. To compensate, the dry bones were replicas of a patient's arthritic tibia and femur, with replica ligaments as well as a surrounding capsule attached and hence were as realistic to a real patient as possible. Fixation of the tracking arm to the bone may also cause inaccuracy if fixation pins bend or loosen due to stress on the fixation point. It is therefore important to design fixtures that are robust and rigid and not loosened (i.e., no movement between the tracking device and the anatomy should be allowed). Furthermore, if after screwing the fixtures, the anatomy of the subject deforms due to overloading of the segment, the possible error as a result of this needs to be evaluated for each robot. In the Sculptor, the tracking arm is quite light, and therefore we assumed stress on the fixation point because the weight of the arm would be minimal.

We acknowledge that during the planning phase, landmarks used to define reference frames are located manually by the surgeon. Srivastava et al. [24] describe the effects of landmark placement variability on kinematic descriptions of the knee. The positions of these landmarks may be open to placement inaccuracy and variability between surgeons. In addition to the accuracy measurements described above, a sensitivity analysis should be performed to determine the likely variability in frame of reference orientations and implant position relative to these introduced by the human operator during planning.

Often in the literature, errors are based on the translational or angular location of the implant and cuts; however, Simon et al. [17] argued that there are ambiguities associated with these data due to a dependence upon the selected coordinate system. It is therefore anticipated in the future for the implant manufacturer to provide a standard protocol for evaluation of location of the implant. The use of dry bones meant that soft tissue balancing could not be recreated and the tibio-femoral angle could not be measured. Although this is an important measure of functional outcome following a UKA, it is widely accepted that component alignment is a major influence on the limb's tibiofemoral angle [24]. In this study, we used a laser scanner instead of CT to find the position of the implant postoperatively since a metallic implant will create artefact in the CT scan and inaccuracy in segmentation. There could also be inaccuracies during segmentation and in CT data itself; however, this is not part of the system and would be operator error, not that of the software.

## 5. Conclusions

Overall our results of segmentation, registration, and cuts made by robot were satisfactory for both components using the Acrobot Sculptor. It is possible to apply the full or part of this protocol in this study in a variety of other products available on the market for better understanding and validation of robotic technology. 

## Figures and Tables

**Table 1:** Translational and rotational error values in UKA implant placement (*n* = 9).

	Tibia						Femoral					
	Translational error (mm)			Rotational error (°)			Translational error (mm)			Rotational error (°)		
	Lateral medial	Anterior posterior	Distal proximal	Flexion extention	Varus valgus	Axial rotation	Lateral medial	Anterior posterior	Distal proximal	Flexion extention	Varus valgus	Axial rotation
Mean	0.0	−0.9	0.8	2.1	−0.8	0.4	0.6	−1.5	−1.1	0.4	−0.5	0.9
SD	1.5	0.5	1.1	0.6	1.6	1.4	0.9	1.4	1.0	3.7	0.9	2.6
Max	1.8	−0.2	2.1	2.2	2.2	3.1	1.9	0.4	−0.1	5.3	0.5	5.3
Min	−2.8	−1.6	−1.7	−3.3	−1.3	−2.4	−1.2	−3.2	−3.5	−4.7	−1.9	−2.1
